# Sweet Wormwood and Tortoise Shell Decoction (Thanh Hao Miet Giap Thang) Induces DNA Damage, S-Phase Arrest, and Apoptosis in MCF-7 Cells via ATR-CHK1 Signaling Pathway

**DOI:** 10.1155/2022/2358290

**Published:** 2022-03-05

**Authors:** Hoang Tam Nguyen Thai, Thuy Vy Nguyen, Thuy Duong Ho Huynh

**Affiliations:** Department of Genetics, Faculty of Biology and Biotechnology, University of Science, VNUHCM, 227 Nguyen Van Cu Street, District 5, Ho Chi Minh City, Vietnam

## Abstract

**Introduction:**

Sweet wormwood and tortoise shell decoction, Thanh Hao Miet Giap Thang (THMGT) in Vietnamese, a traditional formula composed of five ingredients, is used in complementary care in Vietnam for patients who underwent conventional cancer treatment. To expand the clinical use and explore novel functions of THMGT, this study was conducted to investigate the effect of THMGT in terms of antiproliferative activity and selective cytotoxicity toward human breast cancer cells MCF-7.

**Methods:**

Cytotoxicity of THMGT against human breast cancer cells MCF-7 and primary fibroblasts from a heathy donor were studied using sulforhodamine B (SRB) assay. Flow cytometry analysis, immunofluorescence, and western blotting were also performed to elucidate underlying mechanisms of THMGT action.

**Results:**

The SRB assay on treated MCF-7 cells and primary fibroblasts from a heathy donor indicated selective cytotoxicity of THMGT with a selective index of 3.92. Annexin V/PI staining and flow cytometric analysis on stained MCF-7 cells showed that the THMGT-treated cells were arrested at the S phase and subsequently underwent apoptosis. Western blot analysis showed an upregulation of *γ*-H2AX, increased protein levels of phosphorylated CHK1, TP53, and phosphorylated TP53 in a time-dependent manner, and a downregulated expression of ATR and MDM2.

**Conclusion:**

These results suggested DNA damaging effect and ATR-CHK1-mediated cell cycle arrest of THMGT on MCF-7 cells resulting in apoptosis induction.

## 1. Introduction

In countries where traditional medicine is fully integrated in the national healthcare system, as in the case of Vietnam, traditional medical practices, especially herbal drug prescription, could be used as a complementary therapy provided jointly with conventional treatment or as a supportive care in cancer treatment [1–3].

The sweet wormwood and tortoise shell decoction (Qing Hao Bie Jia Tang in Chinese, Thanh Hao Miet Giap Thang (THMGT) in Vietnamese) is a traditional Chinese medicine (TCM) formula, traditionally used to treat late-stage chronic fever diseases. In Vietnam, THMGT was used as a complementary and supportive care for cancer patients. The formula includes five ingredients: Herba *Artemisiae annuae*, Carapax Trionycis, Rhizoma Anemarrhenae, Moutan Cortex, and Radix Rehmanniae. Artemisia species (*A. annua* and *A. apiacea*) are well known for their content of artemisinin that was initially prescribed for antimalarial properties [4]. More therapeutic and preventive effects such as anti-inflammatory, antiangiogenesis, anticancer, and antioxidant were successively attributed to extracts and compounds from these plants [5–7]. Synergistic effects of different active compounds of the herb such as artemisinin, flavonoids, and polyphenols increase the overall biological activities [7]. Carapax Trionycis, the tortoise shell, was obtained from *Tryonix sinensis* Wiegmann. The substance alone, or combined into multi-ingredient formulae, was used for the treatment of endometriosis; it inhibits the proliferation and induces apoptosis in hepatic stellate cells [8–10]. Moutan Cortex, originated from *Paeonia suffruticosa*, was largely used in TCM for the treatment of gynecological disorders [11]. Its extracts have anti-inflammatory, antitumor, antioxidant, and antiplatelet aggregation activities and inhibit migration and metastasis of cancer cells [12–16]. Alone or combined with other herbs, Moutan Cortex has potential as antidiabetic [17, 18] and hepatoprotective agents [19]. Radix Rehmanniae, dried rhizome from *Rehmannia glutinosa*, has anti-inflammatory, antioxidant, antiglycation, and antidiabetic properties [20–24]. Rhizoma Anemarrhenae, the dried rhizome of *Anemarrhena asphodeloides*, has varied biological activities, including anti-inflammatory, anticoagulation, analgesic, antidiabetic, antiallergic, and antitumor [25–30]. Timosaponin AIII, present in aqueous extracts of *A. asphodeloides*, induces cell death by apoptosis [31] and autophagy and has selective cytotoxicity against cancer cells [32, 33]. However, to our best knowledge, the anticancer activity of THMGT has not been elucidated. This study aims to investigate the selective cytotoxicity of THMGT on human breast cancer cells MCF-7 and underlying mechanisms causing tumor cell death.

## 2. Materials and Methods

### 2.1. Preparation of THMGT Decoction

THMGT was composed of five ingredients in the following dosage: Herba *Artemisiae annuae* L (12 g), Carapax Trionycis (*Tryonix sinensis*) (12 g), Rhizoma Anemarrhenae (*Anemarrhena asphodeloides*) (20 g), Radix Rehmanniae (*Rehmannia glutinosa* Libosch) (16 g), and Moutan Cortex (*Paeonia suffruticosa*) (12 g). Carapax Trionycis was listed in Vietnamese pharmacopeia released by the Ministry of Health as Materia medica allowed for use in traditional remedies. The five crude ingredients were obtained from Tue Lan Traditional Medicine and Medical Clinic (HCMC, Vietnam) in January 2016 and identified by Dr. Duc Nghia Nguyen (Tue Lan Traditional Medicine and Medical Clinic). A voucher specimen was deposited at the Department of Genetics, University of Science (HCMC, Vietnam). A total amount of 720 g of all ingredients was quickly washed 2 times with distilled water and soaked in 7.2 L water for 30 min and gently boiled for 3 h to collect the first decoction. Soaking and boiling of ingredients were repeated to obtain the second decoction. The two decoctions were mixed and steamed to reduce to a volume of 720 ml and finally lyophilized to obtain dried powder with amount of 172.8 ± 10.6 g (yield: 24.1 ± 1.5%). The dried powder was dissolved in distilled water to get a final concentration of 100 mg/ml, and the solution was passed through 0.22-*μ*m filter for sterilization.

### 2.2. Chemical Fingerprint Analysis by High-Performance Liquid Chromatography (HPLC)

The dried powder of THMGT was dissolved with HPLC grade methanol (Merck) to a concentration of 10 mg/ml and filtered through a 0.45 *μ*m HPLC syringe filter. Analysis of extracts was carried out using a LC-20AD Shimadzu HPLC system with a PDA-M20A detector. Separation was performed on a C-18 column (150 mm × 4.6 mm, 5 *μ*m; Supelcosil TM, LC-18). The mobile phase consisted of two solvents, double-distilled water (A) and acetonitrile (B). The gradient elution program was set as follows: 0–11 min, 80 : 20 (A : B); 11–25 min, 65 : 35 (A : B); 25–40 min, 55 : 45 (A : B); 40–65 min, 5 : 95 (A : B); 65–70 min, 80 : 20 (A : B); and 66–70 min. The injection volume was 20 *μ*l and the flow rate was maintained at 1.0 ml/min. The column temperature was set at 25°C and the wavelength used for detection was 230 nm.

### 2.3. Cell Line and Primary Fibroblast Culture

MCF-7 cells (HTB-22) were obtained from the American Type Culture Collection (Manassas, Rockville). Cells were grown as monolayer cultures at 37°C and 5% CO_2_, in Eagle's minimal essential medium (E'MEM) supplemented with 10% (v/v) FBS (Sigma), 2 mM L-glutamine (Sigma), 20 mM HEPES (Sigma), 0.025 *μ*g/ml amphotericin B (Sigma), 100 IU/ml penicillin G (Sigma), and 100 *μ*g/ml streptomycin (Sigma). Primary fibroblasts were isolated and identified as previously described [34]. The donor donating tissues gave written informed consent, and this study was approved by the Ethical Committee for Biomedical Research of the Vietnam National University, Ho Chi Minh City (Ref. 1387 QĐ-ĐHQG). Cells were cultured in D'MEM/F12 supplemented with 10% (v/v) FBS, 20 mM HEPES, 0.025 *μ*g/ml amphotericin B, 100 IU/ml penicillin G, and 100 *μ*g/ml streptomycin at 37°C, 5% CO_2_.

### 2.4. SRB Assay

The assay was performed as previously described [34]. Cells were seeded in 96-well plates (SPL Life Sciences) at a density of 1 × 10^4^ cells/well. After 24 h of culture, cells were treated with THMGT at different concentrations for 48 h. Treated cells were fixed with 50% (w/v) cold trichloroacetic acid (Merck) for 1–3 h, washed and stained with 0.2% (w/v) sulforhodamine B (SRB) (Sigma) for 20 min. A protein-bound dye was dissolved in 10 mM Tris-base solution (Promega) after five washes with 1% acetic acid to remove the unbound dye. Optical density values were determined at wavelengths of 492 nm and 620 nm using a 96-well microtiter plate reader (Synergy HT, Biotek Instruments). The percentage of growth inhibition (I%) was calculated according to the formula: I% = (1 − (ODt/ODc) × 100)%, where ODt and ODc are the optical density value of test and control samples, respectively. Camptothecin (CPT) was used as a positive control.

### 2.5. MTT Assay

The assay was performed as previously described [35]. Cells were seeded in 96-well plates (SPL Life Sciences) at a density of 1 × 10^4^ cells/well. After 24 h of culture, cells were treated with THMGT at a concentration of 3 mg/ml for 3, 6, and 9 h. After THMGT treatment, 100 *μ*l of MTT solution (0.5 mg/ml) was added to each well, and the plates were incubated for 4 h at 37°C, and then the media were removed. After dissolving the formazan crystals in 200 *μ*l of DMSO, the absorbance of each plate was measured at 570 nm and 620 nm using a 96-well microtiter plate reader (Synergy HT, Biotek Instruments). The percentage of proliferative inhibition (I%) was calculated according to the formula: I% = (1 − [ODt/ODc] × 100)%, where ODt and ODc are the optical density value of test and control samples, respectively.

### 2.6. Cell Cycle Analysis

MCF-7 cells were cultured for 24 h at a density of 2 × 10^6^ cells per 10 cm dish (SPL Life Sciences). Cells were subsequently treated with 3 mg/ml THMGT for 3, 6, and 9 h. Cells were then collected in Trypsin/EDTA, fixed in cold 70% (v/v) ethanol, and stored at −20°C for at least 24 h. After washing two times with PBS, cells were incubated with 0.2 mg/ml RNase for 30 min at 37°C and subsequently stained with 10 *μ*g/ml propidium iodide staining buffer for 15 min at room temperature. DNA content was analyzed by BD Accuri C6 Plus Flow cytometry (BD Biosciences).

### 2.7. Annexin V/PI Staining for Apoptosis Detection

MCF-7 cells were cultured for 24 h at a density of 2 × 10^6^ cells per 10 cm dish (SPL Life Sciences). Cultured cells were exposed to THMGT at concentration of 3 mg/ml for 3, 6, and 9 h. Annexin V/PI staining was performed according to FITC Annexin V Apoptosis Detection Kit II (BD) protocol. Stained cells were analyzed by BD Accuri C6 Plus Flow cytometry (BD Biosciences). Results were determined as follows: viable cells expressed (−) Annexin V/(−) PI, necrotic cells were (−) Annexin V/(+) PI, early apoptotic cells were (+) Annexin V/(−) PI, and late apoptotic cells were (+) Annexin V/(+) PI.

### 2.8. Western Blotting

MCF-7 cells were lyzed by a RIPA buffer (Thermo Scientific Pierce, USA) containing protease inhibitor cocktail (Complete Protease Inhibitor Cocktail Tablets, Roche Diagnostics GmbH, USA). Lysates were quantitated by the BCA Protein Assay kit (Thermo Scientific Pierce, USA). An amount of 30 *μ*g of total protein samples was analyzed by SDS-PAGE and transferred onto nitrocellulose membranes. After blocking step with a blocking buffer, membranes were incubated overnight at 4°C with primary antibodies. Membranes were subsequently rinsed 5 times with washing buffer (0.1% (v/v) Tween in 1X PBS or 0.1% (v/v) Tween in 1X TBS) and incubated with horseradish peroxidase-conjugated secondary antibody for 1 h at room temperature. Finally, membranes were washed 5 times with washing buffer. Protein signals were visualized by SuperSignal West Pico Chemiluminescent Substrate (Thermo Scientific Pierce, USA) and scanned by ImageQuant LAS 500 (GE Healthcare Biosciences, UK). Antibodies used for western blot in this study are listed in Supplementary Table S1. *β*‐Actin was used for normalization, and the protein expression was evaluated relative to the nontreated cells at each time point.

### 2.9. Immunofluorescence Microscopy

Cells were seeded at a density of 1.5 × 10^5^ cells per 22 × 22 mm glass coverslip (Duran) and cultured for 24 h at 37°C and 5% CO_2_. Cells were subsequently exposed to 3 mg/ml THMGT for 3 h. Coverslips were washed with 1X PBS (-), fixed with 4% (w/v) paraformaldehyde in 10 mins, incubated in TBS 1X 0.3% (v/v) Triton X-100 for 20 min, blocked in 0.2% (w/v) BSA in 60 min at room temperature with shaking, and incubated with *γ*-H2AX antibody (sc-517348, 1 : 200 dilution) overnight at 4°C in a humid chamber. After being washed with TBS 1X 0.1% (v/v) Tween-20 and incubated with Alexa 488 anti-mouse antibody for 60 min at room temperature in a humid chamber, coverslips were washed with TBS 1X 0.1% (v/v) Tween-20 and incubated with 10 *μ*l Hoechst (1 *μ*g/ml) for 10 min at room temperature. Finally, coverslips were mounted and observed on a fluorescence microscope. For each condition, at least 100 cells from three independent experiments were scored, and cells with more than four foci per nucleus were graded as positive.

### 2.10. Statistical Analysis

Data were represented as means ± standard deviation (SD). Statistical analyses were performed using a two-tailed Student's *t* test. Results were considered statistically significant at *p* value < 0.05.

## 3. Results

### 3.1. Chemical Fingerprint of the THMGT Formula

**Figure 1:**
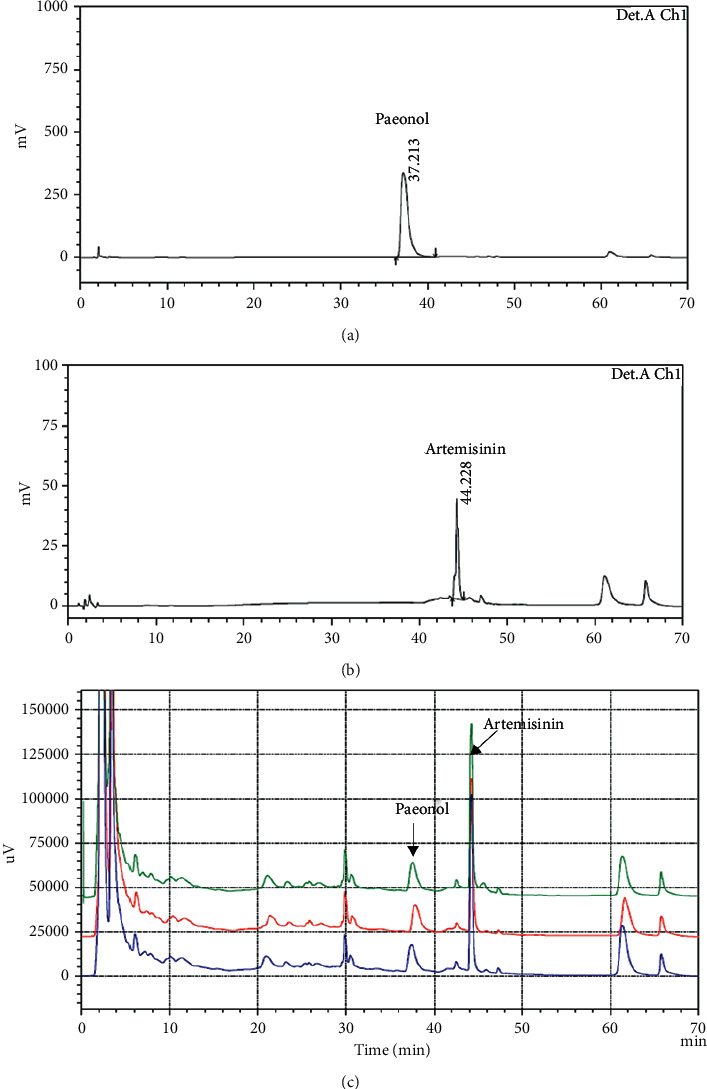
HPLC fingerprint chromatogram of THMGT. (a, b) Chromatogram of the two standard compounds, paeonol and artemisinin, respectively. (c) HPLC chromatograms of different THMGT decoctions have similar patterns.

The HPLC fingerprint chromatogram of THMGT is shown in Figure 1. Paeonol and artemisinin were well identified in THMGT by comparing both retention times and UV spectra. Our results also showed that HPLC chromatograms of THMGT decoctions have similar patterns, suggesting reproducibility in the preparation technology and the quality of the medicinal materials (Figure 1(c)).

### 3.2. THMGT Exerted Selective Cytotoxicity in MCF-7 Cells

**Figure 2:**
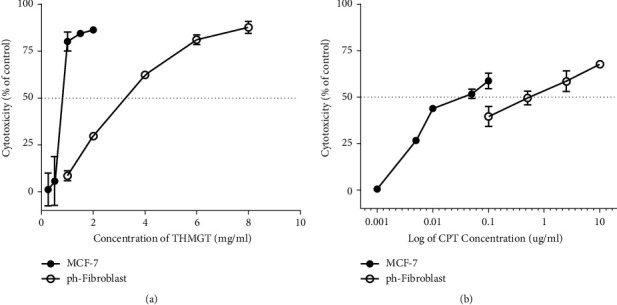
Cytotoxicity of THMGT on MCF-7 cells and primary human fibroblasts from healthy donor (ph-fibroblast). (a) MCF-7 cells and ph-fibroblasts were treated with THMGT at different concentrations for 48 h. Cytotoxicity was detected by the SRB assay. (b) MCF-7 cells and ph-fibroblasts were treated with CPT as a positive control at different concentrations for 48 h. Cytotoxicity was detected by the SRB assay. Data are shown as mean ± SD of three independent experiments.

An ideal anticancer compound is one that has selective cytotoxicity toward tumor cells with no or less toxicity against normal cells. Selective cytotoxicity was measured through the selective index (SI). The more the SI value of a compound is great, the more it displays selective cytotoxicity. An SI value less than 2 indicates nonselectivity. We assessed the selective cytotoxicity of THMGT through calculation of its SI. The SI value was determined as the ratio between IC_50_ values of the formula for human primary fibroblasts and for human breast cancer cells MCF-7. The fibroblasts and MCF-7 cells were treated with the same serial dilution of THMGT and subjected to SRB assay to determine IC_50_ values (Figure 2). IC_50_ values of THMGT for MCF-7 cells and fibroblasts were 0.79 ± 0.08 and 3.06 ± 0.11 mg/ml, respectively, giving a SI of 3.92 ± 0.43. As a positive control, IC_50_ values of CPT for MCF-7 cells and fibroblasts were 0.10 ± 0.01 and 0.54 ± 0.17 *μ*g/ml, respectively, giving a SI of 5.13 ± 1.14.

### 3.3. THMGT Executed Its Selective Cytotoxic Effects through Induction of DSBs in MCF-7 Cells

**Figure 3:**
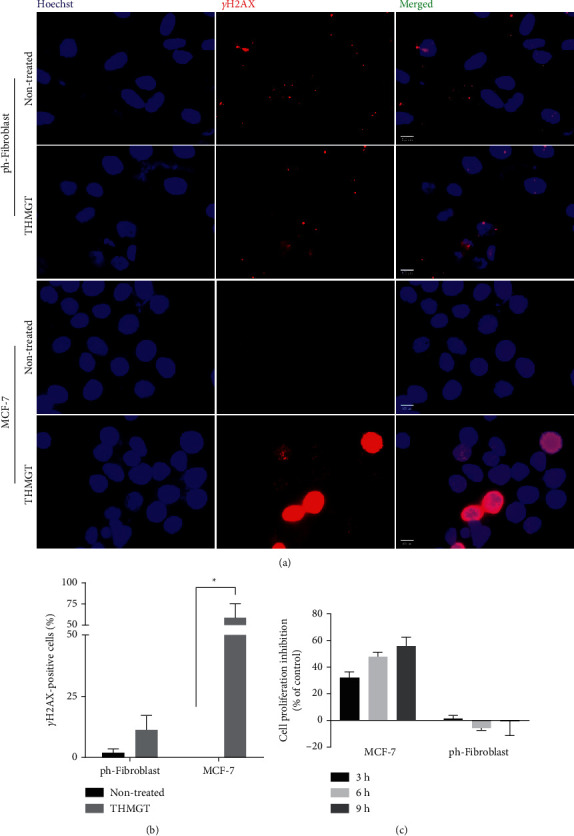
DSB accumulation in MCF-7 cells treated with THMGT. (a) Immunofluorescence staining of *γ*-H2AX foci in MCF-7 cells and ph-fibroblasts. Cells were treated with THMGT at a concentration of 3 mg/ml for 3 h and stained with a primary antibody against *γ*-H2AX and FITC-conjugated secondary antibody. Nuclei were counterstained with Hoechst. The data presented are representatives of three independent experiments. (b) Quantitation of the immunofluorescence staining data obtained from three independent experiments is shown ( ^*∗*^*p* < 0.05). (c) Antiproliferative effects of THMGT on MCF-7 cells and ph-fibroblast. Cells were treated with THMGT at a concentration of 3 mg/ml for 3, 6 and 9 h and the proliferation was determined by the MTT assay. Data are shown as mean ± SD from at least three independent experiments.

Several anticancer drugs selectively kill cancer cells by inducing DNA damage; the genomic instability and DNA repair defects of cancer cells make them more vulnerable than normal cells to the cytotoxicity of DNA-damaging agents [36]. Among DNA lesions, DSB is considered to be the most severe form of DNA damage. To elucidate the possible mechanisms of THMGT-induced selective cytotoxicity, we examined the accumulation of DSB in THMGT-treated MCF-7 cells through the formation of *γ*-H2AX. Several studies have demonstrated that phosphorylation of histone H2AX, called *γ*-H2AX, is specific for DSB formation and not formed in other cellular DNA damage [37]. Compared with untreated cells, THMGT treatment at a concentration of 3 mg/ml increased H2AX phosphorylation after 3 h, as determined by immunofluorescence staining analysis. Furthermore, THMGT did not induce DSB formation in human primary fibroblasts (Figures 3(a) and 3(b)). In addition, the result of MTT assay showed that the proliferation level of MCF-7 cells treated with THMGT was diminished to 60% in comparison with the nontreated cells after 9 h, while the proliferation of the human primary fibroblasts was not affected (Figure 3(c)). These results indicate that THMGT selectively induces DSB lesions, thereby causing an antiproliferation in human breast cancer cells MCF-7.

### 3.4. THMGT Induced Cell Cycle Arrest at the S Phase in MCF-7 Cells

**Figure 4:**
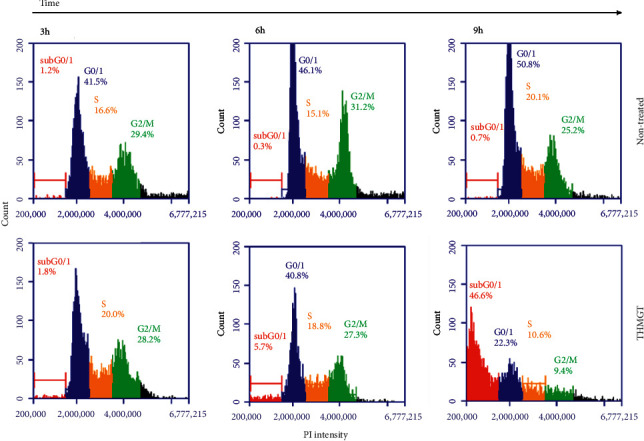
THMGT induced cell cycle arrest at the S phase. MCF-7 cells were treated with THMGT at concentration of 3 mg/ml for 3, 6, and 9 h PI staining of the fixed cells was performed to obtain the flow cytometry-based cell cycle distribution profiles of the analyzed cells. The cell cycle phases are depicted in each panel.

To understand whether THMGT inhibits the proliferation of MCF-7 cells through a cell cycle specific or nonspecific effect, flow cytometric analysis of cell cycle distribution based on DNA content was performed on PI-stained MCF-7 cells exposed to THMGT at a concentration of 3 mg/ml for 3, 6, and 9 h. Significant augmentation of the cell population in the S phase of treated MCF-7 cells compared to nontreated cells was observed at 3 and 6 h treatment, indicating a S-phase arrest effect of THMGT on MCF-7 cells. After 9 h exposure to THMGT, half of the cell population was found in the sub-G1 area (Figure 4 and Table 1). These results suggest that the S-phase arrest observed at 3 and 6 h treatment with THMGT resulted in apoptosis induction in MCF-7 cells, an event evident at 9 h time point.

### 3.5. THMGT Induced Apoptosis in MCF-7 Cells

**Figure 5:**
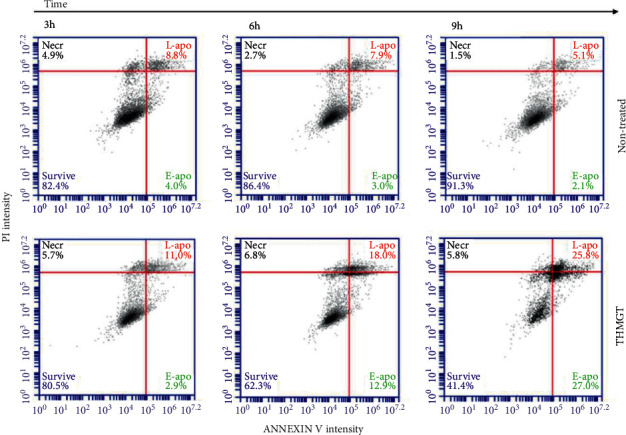
THMGT induced the apoptosis of MCF-7 cells. MCF-7 cells were treated with THMGT at a concentration of 3 mg/ml for 3, 6, and 9 h Annexin V/PI staining was performed to detect apoptosis. The lower right quadrant of the fluorescence activated cell sorting (FACS) output indicates the percentage of early apoptotic cells (Annexin V-stained cells), while the upper right quadrant indicates the percentage of late apoptotic cells (Annexin V+/PI-stained cells).

Apoptosis induction is a common mechanism of action of numerous anticancer compounds. To investigate the apoptosis induction capacity of THMGT, MCF-7 cells were stained with PI and Annexin V and subsequently analyzed by flow cytometry after treatment with or without THMGT at a concentration of 3 mg/ml for 3, 6, and 9 h. Survival cells after treatment exhibited no staining; cells at early apoptosis stage were positive with Annexin V staining whereas late apoptotic cells were stained with both Annexin V and PI. Necrotic cells only displayed PI staining. As shown in Figure 5 and Table 2, compared to nontreated cells, the survival fraction of MCF-7 cells after 3, 6, and 9 h treatment with THMGT decreased progressively from 76% to 18% of total cells. The fraction of apoptotic cells increased with treatment time, with late apoptotic stage cells exhibiting a striking augmentation to reach 64% after 9 h treatment compared to 10% at 3 h treatment. These results clearly indicate an apoptosis induction effect of THMGT on MCF-7 cells, beginning before the 6 h time point.

### 3.6. THMGT Activated Checkpoint Kinase 1 (CHK1)-Mediated DNA Damage Response (DDR), While Inhibited the Expression of ATR Protein

**Figure 6:**
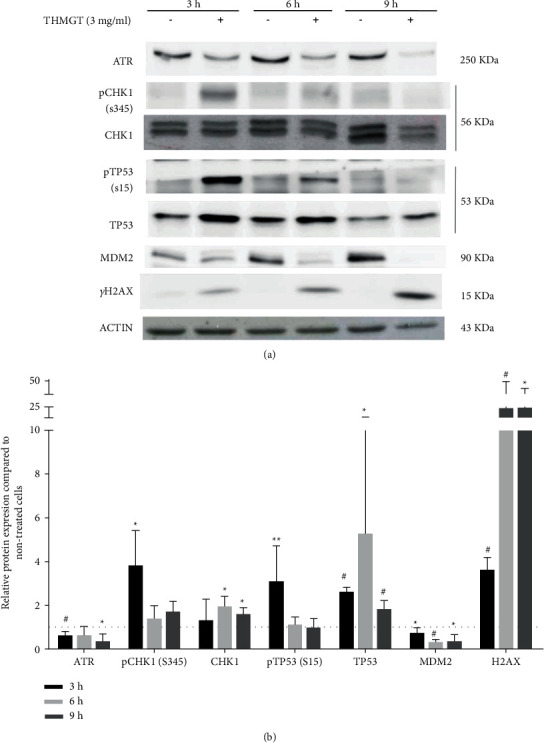
THMGT triggered DDR in MCF-7 cells. (a) The cells were treated with THMGT at a concentration of 3 mg/ml for 3, 6, and 9 h Collected cells were analyzed for the expressed levels of ATR, pCHK1, CHK1, pTP53, TP53, MDM2, and *γ*-H2AX by western blot. *β*-Actin expressions were measured as internal controls to show equal protein loading. The data presented are representatives of three independent experiments with similar results. (b) Relative protein level compared the nontreated cells at each time point ( ^*∗*^*p* < 0.05, ^#^*p* < 0.1).

To further reveal the mechanism of THMGT inducing DSBs, leading cell cycle arrest at the S phase and apoptosis in MCF-7 cells, we analyzed the expression levels of DNA damage signaling molecules, including *γ*-H2AX, ATR, CHK1, TP53, and MDM2, and the phosphorylation of CHK1 at S345 and TP53 at S15. Analyses of proteins that contributed to DNA damage response in MCF-7 cells exposed to 3 mg/ml THMGT showed that the levels of *γ*-H2AX, an indicator of DNA damage, were markedly increased compared to nontreated cells for all the three time points. The ATR expression was progressively reduced since the 3 h time point and was not observable at 9 h after treatment. We observed a marked increased signal for the S345 phosphorylated form of CHK1 (pCHK1) at the 3 h time point, followed by dramatic signal decreases at 6 and 9 h time points. The CHK1 expression in MCF-7-treated cells was increased compared to control cells at 3 h time point, and unchanged compared to control cells for two remain time points investigated. Both TP53 and its phosphorylated form at S15 (pTP53) also expressed a sharp upregulation in MCF-7 cells at 3 h time point followed by a progressive time-dependent downregulation. The expression of TP53 and especially TP53 phosphorylated at S15 in MCF-7 cells showed an important increase at 3 h time point compared to control cells. This increased expression was maintained at 6 h time point though lower than that at 3 h time point. A decreased expression of MDM2, the negative regulator of p53, was observed for the three time points (Figure 6). These results suggest that THMGT induces DSB accumulation and consequently causes DDR through the ATR pathway in MCF-7 cells.

## 4. Discussion

TCM utilizes a holistic approach based on multicomponent drugs to address different targets in the body. In cancer treatment, herbal formulations based on combinations of ingredients are largely used. In Vietnam, THMGT is a formula used for supportive care in cancer treatment for patients who underwent chemotherapy or radiotherapy. So far, different ingredients from THMGT have been demonstrated to have anticancer activity [7, 8, 14, 23, 31]. To expand the clinical use and explore novel functions of THMGT, we evaluated the antiproliferative and apoptosis-induction effects of the formula on cancer cells, which have not been elucidated yet. Our results demonstrated that THMGT triggered DSB accumulation, ATR-CHK1-mediated S-phase arrest, and ultimately apoptotic death in human breast cancer cells MCF-7 with minimal effects on cultured primary fibroblasts from a healthy donor.

We observed that MCF-7 cells expressed a *γ*-H2AX accumulation in only 3 h after THMGT exposure denoted DSBs as the main DNA lesions. At the same time point, we observed a significant increase proportion of S-phase cells that indicated an S-phase arrest. The S-phase arrest could occur as an independent event from DDR or as the consequence of dramatic unresolved DNA damage.

Human cells respond to DNA damage by inducing checkpoint pathways to arrest cell cycle progression, giving opportunities for DNA repair machinery to proceed. The first step in the DDR consists of sensing the DNA lesions. The “sensors” of the checkpoints are ATM and ATR kinases that subsequently activate “effector” kinases CHK2 and CHK1, respectively. Depending on the type of lesion and cell cycle phase it occurs, different pathways are involved. It is proposed that the ATM-CHK2 pathway controls the G1 checkpoint and the ATR-CHK1 pathway controls the S and G2/M checkpoints [38]. Furthermore, ATR monitors a replication fork progression and is responsible for maintaining replication fidelity. Checkpoint activation is triggered when replication forks are stalled by abnormally structured or damaged DNA [39]. In this study, THMGT-treated MCF-7 cells showed increased CHK1 phosphorylation at S345 at 3 h time point, followed by striking signal decreases at 6 and 9 h time points, while the protein expression was unchanged compared to control cells for the three time points investigated. The phosphorylated CHK1, which in turn induced phosphorylation of TP53 at S15 in the treated MCF-7 cells at 3-h time point that triggered an S checkpoint, and the consequent S-phase arrest under a DNA damage stimulus. Notably, we observed that the expression of ATR protein in MCF-7 cells was progressively reduced since 3 h time point and was not observable at 9 h after treatment with THMGT. CHK1 is rapidly phosphorylated at S317 and S345 by ATR in response to DNA damage. Therefore, the initial occurrence of phosphorylated CHK1 at S345 observed in this study suggested an early induction of ATR-mediated DDR by THMGT. However, THMGT treatment decreased the expression of ATR in MCF-7 cells and thus caused the subsequent reduction in phosphorylated CHK1 abundance, implicating an attenuated DDR. Indeed, nine hours after THMGT exposure, histone H2AX molecules remained highly phosphorylated in the MCF-7 cells compared with nontreated cells suggesting delayed DSB repair or accumulation of unrepaired DNA damage. It has been reported that, in the absence of either ATR or CHK1, mammalian cells rapidly lose viability, most likely because of a disruption in DNA replication fidelity, ultimately resulting in apoptotic death [40, 41]. These might explain the early accumulation of S-phase MCF-7 cells followed by rapid apoptosis process observed in the above-mentioned flow cytometric analysis. Selective cytotoxicity toward cancer cells with minimal harm to normal cells is an ideal approach in cancer treatment. In normal cells, DNA lesions activate ATM-CHK2 and/or ATR-CHK1 pathways that mediate G1/S, S, and G2/M checkpoints to arrest cell cycle progression for DNA repair. Many cancers exhibit loss of G1/S checkpoints through TP53, ATM mutations, or Rb loss. This results in that the cancer cells rely on later checkpoints for DNA repair and cell survival, including the ATR-CHK1 pathway. If the ATR-CHK1 pathway is suppressed in G1/S checkpoint-deficient cells in the presence of DNA lesions, cells will not trigger cell cycle arrest for DNA repair, leading to cell death. Thus, inhibiting the ATR-CHK1 pathway has been an attractive strategy to selectively sensitize G1/S checkpoint-deficient cancer cells to therapies that damage DNA, without compromising normal cells with proficient the G1/S checkpoint [38]. Moreover, it is demonstrated that a slight reduction of functional ATR was sufficient to be lethal in oncogenic tumors while sparing normal bone marrow and intestinal cells [42]. Therefore, in this study, the translational downregulation of ATR, a couple with the induction of cellular DNA damage might contribute to selectively targeting MCF-7 cells as well as potentially other cancer cells by THMGT in future investigations.

THMGT includes five ingredients, Herba *Artemisiae annuae*, Carapax Trionycis, Rhizoma Anemarrhenae, Moutan Cortex, and Radix Rehmanniae. Previous studies have proven that artemisinin and timosaponin AIII, major active components of *Artemisia annua* and *Anemarrhena asphodeloide*, respectively, were capable of inducing DNA damage in cancer cell lines [43, 44]. Particularly, artesunate, an artemisinin's derivative, provoked a DDR with phosphorylation of ATM, ATR, CHK1, and CHK2 in LN-229 glioma cells after 8-hour treatment [45], while timosaponin AIII activated DDR through the ATM-CHK2 pathway in MCF-7 cells after treatment for 10–20 min [44]. These compounds have been demonstrated arresting a cell cycle at either the G1 or G2/M phase, and subsequently triggering apoptosis in tumor cells [44, 46]. Furthermore, according to the HPLC analysis, artemisinin and paeonol are the major components of THMGT. However, paeonol, an active ingredient in Moutan cortex, shows anti-inflammatory, antitumor, and antioxidant properties and inhibits migration and metastasis of cancer cells based on current report [12–16]. Therefore, the sustained DSB formation and S-phase arrest observed in THMGT-treated MCF-7 cells at least partially resulted from a combined effect of two ingredients: Artemisia *annua* and *Anemarrhena asphodeloide*. Interestingly, to the best of our knowledge, none of individual THMGT components has been shown to downregulate the expression of ATR leading to attenuated DDR. This emphasizes the use of a mixture of ingredients in traditional medicine preparation, resulting in increasing the effectiveness of herbal remedies due to synergistic effects. Future investigations will evaluate the relationship between the THMGT ingredients and the ATR-CHEK1 signaling pathway.

Although not essential for the repair of the majority of DSBs, the ATM activity is required for the repair of a subset of DSB generally associated with heterochromatin [38]. Previous studies have shown that the ATM gene was disrupted in the human breast cancer cell line MCF-7 [47]. Targeting DNA damage checkpoint kinase, such as ATR/ATM, could be promising for cancer treatment. Therefore, it is necessary to conduct more in-depth studies to comprehensively understand whether THMGT has any effect on the DDR signaling pathway ATM-CHK2.

In conclusion, the traditional remedy THMGT expressed high selective cytotoxicity and antiproliferation on MCF-7 cells toward human primary fibroblasts from a healthy donor. The cytotoxicity and antiproliferation of THMGT relied on its capacity of simultaneous inducing DNA damage and DDR attenuation contributed to the S-phase arrest, and ultimate apoptosis. These results supported the potential utility of this formula as complementary healthcare for chemotherapy and radiotherapy. Further studies to clarify mechanisms underlying the selective antiproliferation and to predict the optimal timing of drug administration are needed.

## Figures and Tables

**Table 1:** Cell cycle analysis of MCF-7 cells exposed to 3 mg/ml THMGT for 3, 6, and 9 hours.

Phase	Proportion of cell population (%) determined at different time points					
	3 h		6 h		9 h	
	Nontreated	THMGT	Nontreated	THMGT	Nontreated	THMGT
sub-G1	1.0 ± 0.1	1.2 ± 0.5	0.6 ± 0.6	2.3 ± 3.0	1.5 ± 1.0	50.8 ± 8.8^*∗∗*^
G1	38.7 ± 3.4	40.3 ± 3.7	47.9 ± 6.1	39.9 ± 3.0	45.1 ± 4.9	20.4 ± 2.1^*∗∗*^
S	17.3 ± 0.6	23.1 ± 2.8^*∗∗*^	15.5 ± 1.9	19.4 ± 0.6^*∗*^	16.7 ± 4.0	11.5 ± 4.3
G2/M	33.7 ± 3.2	30.3 ± 2.8	27.4 ± 5.4	30.7 ± 2.9	29.6 ± 4.1	11.4 ± 4.1^*∗∗*^

Statistical analyses were performed using a two-tailed Student's *t*-test. ^*∗∗*^*p* < 0.01; ^*∗*^*p* < 0.05. Data are shown as mean ± SD of three independent experiments.

**Table 2:** THMGT induced the apoptosis of MCF-7 cells.

	Proportion of cell population (%) determined at different time points					
	3 h		6 h		9 h	
	Nontreated	THMGT	Nontreated	THMGT	Nontreated	THMGT
Survive	84.1 ± 4.7	80.5 ± 10.7	82.5 ± 3.4	60.3 ± 7.9^*∗*^	90.0 ± 5.3	44.1 ± 2.8^*∗∗*^
E-apo	6.9 ± 3.8	8.0 ± 9.5	5.4 ± 4.1	8.0 ± 4.8	4.3 ± 4.8	15.6 ± 13.3
L-apo	7.1 ± 2.1	9.1 ± 3.2	8.8 ± 1.5	26.3 ± 10.4^*∗*^	4.3 ± 1.5	33.9 ± 7.8^*∗∗*^
Necrosis	1.9 ± 2.6	2.4 ± 2.9	3.3 ± 2.6	5.4 ± 2.7	1.4 ± 0.6	6.4 ± 4.1

E, early; L, late; statistical analyses were performed using a two-tailed Student's *t*-test. ^*∗∗*^*p* < 0.01; ^*∗*^*p* < 0.05. Data are shown as mean ± SD of three independent experiments.

## Data Availability

The data used to support the findings of this study are included within the article and the supplementary information file.

## Supplementary Materials

Supplementary Materials Table S1: antibodies used for western blot.
