# Supplementary material for: Sweet Wormwood and Tortoise Shell Decoction (Thanh Hao Miet Giap Thang) Induces DNA Damage, S-Phase Arrest, and Apoptosis in MCF-7 Cells via ATR-CHK1 Signaling Pathway
Source: Evid Based Complement Alternat Med. 2022 Mar 5;2022:2358290. doi: 10.1155/2022/2358290 (PMC8917953; doi:10.1155/2022/2358290)
Supplement: Supplementary Materials — Table S1: antibodies used for western blot. [file 2358290.f1.docx]

**Table S1. Antibodies used for Western blot**

| **Antibody** | **Code/Company** | **Final conc. dilution** |
| --- | --- | --- |
| anti-ATR | Sc 1887/ Santa Cruz | 1:500 |
| anti-MDM2 | OP46/ Calbiochem | 1:500 |
| anti-TP53 | sc 126/ Santa Cruz | 1:500 |
| anti-pTP53 (S15) | 9284/ Cell Signaling | 1:1000 |
| anti-pCHK1 (S345) | 23485/ Cell Signaling | 1:1000 |
| anti-CHK1 | Sc 8408/ Santa Cruz | 1:500 |
| anti-γH2AX | Sc 517348/ Santa Cruz | 1:500 |
| anti-Actin | Sc 47778/ Santa Cruz | 1:1000 |
| anti-Goat HRP | Sc 2020/ Santa Cruz | 1:10000 |
| anti-Mouse HRP | Sc 2005/ Santa Cruz | 1:10000 |
| anti-Rabbit HRP | Sc 2313/ Santa Cruz | 1:10000 |
| anti-Rat HRP | Sc 2006/ Santa Cruz | 1:10000 |
